# Migration choices of China’s older adults and spatial patterns emerging therefrom (1995–2015)

**DOI:** 10.1371/journal.pone.0290570

**Published:** 2023-08-24

**Authors:** Rongwei Wu, Linguo Wu

**Affiliations:** 1 Population Development and Policy Research Center, Chongqing Technology and Business University, Chongqing, China; 2 School of Geography and Planning, Sun Yat-sen University, Guangzhou, China; 3 School of Law and Sociology, Chongqing Technology and Business University, Chongqing, China; Guangzhou Institute of Geography, Guangdong Academy of Sciences, CHINA

## Abstract

The steady increase in China’s ageing population and an upswing in migration among the country’s population, on the whole, has caused a continuous expansion of the scale of older migrants. The migration of older adults not only directly affects the well-being of individual families but also significantly impacts the population structure and economic development of the places of origin and destination. Despite this, in China, the various relevant aspects concerning this age group and, in particular, its migration choices and the patterns thereof have only rarely been the subject of sound research. The study presented in this paper seeks to fill this gap; the present study makes use of the microdata obtained from the national population censuses of 2000 and 2010 and the 1% population sample surveys conducted nationally in 2005 and 2015. The findings of the present study were the following: ① During 1995–2015, the efficiency of older adults’ migration was significantly higher in the eastern region than in the central and western regions. ② Older individuals migrating to urban areas are increasingly choosing, for their relocation, economically developed, urban areas such as the Yangtze River Delta, Pearl River Delta and Beijing-Tianjin-Hebei region. ③ Relocation of older adults to urban areas was much more than to rural areas. The latter group has a more diverse choice of destination, and the larger migration flow is primarily from developed provinces to relatively underdeveloped provinces. ④ The results of binary logit regression indicated that the factors that significantly and consistently influence the migration decisions of older adults were found to be the following: age, education level, health status, the primary financial resource, children aged ≤ 6 years being members of the household that would receive the migrants, and the average wage of employees. As for the geographical characteristics of the province to which the older adults migrate, a substantial difference was observed between the preferences of older adults migrating to urban regions and those of older adults relocating to rural areas. The findings of the present study provide further insight into the decision-making of older adults regarding migration. Further, these findings constitute an empirical basis for the local governments concerned to devise and implement policies to better cope with an ageing population.

## 1 Introduction

Western nations began gradually transitioning into an ageing society from the late 19th century through the first half of the 20th century. The willingness to migrate becomes a trend, among individuals as they get older, growing diversity of locations to migrate to and an increased awareness of relocation options in old age account for this trend. Older adults’ migration has several interconnected implications. It directly alters the regional population’s age structure and changes how adults are spatially distributed in an area [1]. However, migration also makes rebuilding social networks more difficult for older adults, thereby heightening their sense of loneliness [2, 3]. On the other hand, it can inject additional capital into the existing economic base of the destination, promote the development of local industries, such as pension and healthcare, and attract a considerable number of young people to work in the service industry, thus creating a large number of jobs. Understanding the choices and spatial patterns concerning older adults’ migration, in China, along with the factors influencing them is fundamental to explaining their comprehensive impact on individuals and regions.

A review of the extensive literature on this topic reveals that the spatial patterns of older adults’ migration can be simply divided into the following two basic types: ‘centripetal migration’ and ‘centrifugal migration’ [4–6]. ‘Centripetal migration’ refers to migration from rural areas to cities. This type of migration pattern is very appealing to older adults who seek an urban lifestyle or wish to live with their children and young grandchildren. By providing high-quality, easily available healthcare, a diverse range of cultural and recreational opportunities, accessible public amenities, and more frequent intergenerational interactions with out-migrating children and grandchildren, large cities help older citizens realise their desire for good health and a sense of belonging [7–9]. ‘Centrifugal migration’ refers to the movement away from metropolitan areas to less urbanised or rural areas that have a pleasant climate and better living facilities [10–12]. ‘Return migration’ is the most common type of ‘centrifugal migration’. Older adults who were born and/or who grew up in rural areas are more likely to return to their place of birth or upbringing; their early-life experiences greatly influence their return migration–related decisions [13]. Such attachment to a place is defined as ‘a positive emotional bond between an individual and a specific place, characterised by the individual’s tendency to remain close to that place’ [14].

The spatial patterns of older adult migration vary significantly among countries. In developed countries such as the United States (US) and France, this pattern is primarily ‘centrifugal’, that is, the older adults tend to move from urban centres in colder regions to small coastal towns and villages with warmer weather, which has a ‘sunny’ characteristic. The colder weather in the Northern and Midwestern US has caused approximately 60% of the older adults there to migrate after their retirement to the Sun Belt, i.e. to places like Florida and California [15]. Schaffar’s study of the migration patterns of older adults in France also concluded that retirees choose to move long distances even to relocate to destinations that are sunny and pleasant [12]. However, the tendency to move to sunnier areas has gradually ‘cooled off’ in recent years [16]. In Poland, for instance, ‘centripetal migration’ to cities is the primary trend, with the annual urban migration in Poland during 1990–2017 being approximately twice as much as the annual rural migration [17]. The proportion of older people born in the 1920s and 1930s who migrated to urban centres was 19.6% and 21.5%, respectively, while the proportion of older people migrating to the suburbs was only 8.2% and 5.7%, respectively [18].

The migration pattern of China’s older adults is similar to that of older adults in Poland; however, the underlying reasons for this similarity are quite different. Traditional Chinese society is influenced by Confucianism, wherein interpersonal networks are built based on what is referred to as ‘blood relations’ or a family dependency network centred on spouses and children [19, 20]. The vast majority of China’s present-day working-age population was born in the late 1970s, under the one-child policy, and being a well-educated generation, it is seeking employment in large cities, for they are mostly attracted by a fully developed urban labour market. When the one-child generation settles down in big cities, marries, and has children, their parents usually choose to relocate to take care of their grandchildren because the new parents are too busy to do so all by themselves. Caring for their grandchildren enables older adults to experience a sense of value, mitigates the risk of loneliness and depression, helps them maintain an active way of life, improves their cognitive functions, and enhances their mental health and life satisfaction [21].

Compared with studies in developed countries that have focused on older adults and have developed theories and models by drawing on many perspectives [22, 23], the present study focusing on older adults in China is still focused on the ‘migrating population’ among them. Many studies have been conducted to identify the spatial patterns concerning migration in China and the factors influencing these patterns; these studies have relied on different data sources and methods. On the whole, China’s migrants are predominantly of working age, and they are primarily driven by economic considerations that underlie their keenness to move from less developed rural areas to economically developed mega-cities in search of better employment opportunities [24–27]. Since barriers to household registration are faced in most of China’s cities, achieving household registration after migration is often difficult for older adults. As they get older, the health status of these adults deteriorates, and the complicated dual urban-rural household registration system leaves out the older adult migrants because they cannot obtain household registration. This makes them vulnerable and prone to facing problems such as lack of social security and inability to avail of medical insurance reimbursements in places other than their place of origin., This only increases their health risks [20]. Although a large number of studies from other countries can provide valuable insights and ideas for the study of China’s older migrants, given the obvious differences in social systems, the experience of other countries would not be entirely applicable to the Chinese context. On the one hand, unlike foreign older adults who are influenced by the events in their life, such as retirement, physical decline, and widowhood, China’s older adults are influenced by traditional Confucianism and observe a family-oriented ethic. Further, the intergenerational life course exerts a greater impact on older adults’ decisions to migrate in later life. On the other hand, the vast majority of older adults in Western countries who have had jobs and have benefitted from a retirement system are significantly more likely to migrate after retirement in search of a more liveable and attractive retirement destination. However, as the majority of China’s older adults are located in rural areas, get no benefit from a retirement system, and have little savings, the differences in the study subjects will inevitably lead to differences in their behavioural characteristics. It is particularly important to recognise and understand the characteristics of older adults’ migration behaviour with due regard to the Chinese context and the factors that influence this population’s decision-making.

To expand the scope of research concerning migration among China’s older adults and identify the characteristics that are specific to urban and rural migrants, certain basic information must be available for exploring the impact that migrant older adults have on their families and societies in the areas of their origin and destination. The literature review carried out for this study is summarised in the initial sections of this paper, which also introduce the experience and background of migrant older adults. These sections also detail the basic demographic characteristics of this population in China and self-reported causes of their in-country migration by referring to microdata from the 2000 and 2010 national censuses and the 2005 and 2015 1% national population sample surveys. Subsequently, in the context of the unique urban–rural dichotomy in China, this paper analyses the differences in the spatial patterns of migration of older adults to urban and rural areas. It also discusses the factors that influence the choice to migrate and location-related decisions of older adults in China and how they manifest at three levels: individual, family, and regional. This paper then discusses the present study’s findings, expounds upon their significance, and touches upon the future prospects of older adult migration in China for further research on this topic.

## 2 Materials and methods

### 2.1 Data source

The population data used in this study were mainly obtained from the microdata sample of the fifth and sixth national censuses in 2000 and 2010 and the 1% national population sample surveys in 2005 and 2015. The actual older migrant data are derived from statistical data according to the corresponding sampling ratio each year. Due to the small sample size of the older adults’ migration the Tibet, as well as the lack of data from Hong Kong, Macau, and Taiwan, none of them were included in the study. Therefore, this study takes 30 provincial administrative units in China as the study area, including 22 provinces, 4 autonomous regions, and 4 municipalities directly under the central government.

To prevent the role of the time factor, i.e., simultaneity bias, in influencing older migrants, all provincial variables in this study are year-end data for 1995, 2000, 2005, and 2010, taken mainly from the ‘China Statistical Yearbook’. Among them, Chongqing was established as a municipality directly under the Central Government in 1997, and the district includes the former Chongqing, Wanxian, Fuling, and Qianjiang areas of Sichuan Province. Therefore, the data of Chongqing in 1995 are combined according to the data of Chongqing, Wanxian, Fuling, and Qianjiang areas in the ‘Sichuan Statistical Yearbook 1996’. The distance data were obtained from the ‘National Railway Passenger Train Timetable’ using the minimum time of railroad transportation between provincial capitals. All variables are taken as natural logarithms in the operation to facilitate the elasticity interpretation of the effect estimates later.

Finally, the National Bureau of Statistics’ statistical zoning and urban-rural categorization codes were used to classify the existing residences of older adults into urban and rural categories. The urban-rural categorization codes 111, 112, 121, 122, and 123 are situated in urban areas, whereas 210 or 220 are situated in rural regions.

### 2.2 Definition of concepts

#### 2.2.1 Migrating population

Migration is a type of population movement, which entails the physical transfer of people from geographical area to another. Such movement usually involves a long-term, if not a permanent, change in the residence of the individuals concerned from the place of departure to the place of entry. The present study defines older migrants as Chinese citizens whose residence changed across provincial administrative units at the time of the census (November 1, 2000, 2005, 2010, and 2015) and the census that was carried out five years before that (November 1, 1995, 2000, 2005, and 2010).

#### 2.2.2 Older adults

The term ‘older adults’ refers to those who have reached a certain age limit, which is typically 60 or 65 years or more. Since the migration behaviour of older adults usually occurs after their retirement [22, 28], this study takes the age of 60 years as the demarcation line for classifying older adults according to the categorisation of the current retirement age of workers in the *Interim Measures of the State Council on the Resettlement of Old*, *Weak and Disabled Cadres* and the *Interim Measures of the State Council on the Retirement and Retirement of Workers in China*.

### 2.3 Methods

#### 2.3.1 Binary logit model

The decision of older adults to migrate is based on two options: ‘migration’ and ‘non-migration’, which can be transformed into a dummy variable of ‘1’ or ‘0’. An econometric approach, based on the binary choice theory is commonly employed to analyse this type of problem, namely the binary logit model, wherein the dependent variable of the model is a binary variable, expressed in the form of the following equation:

$$p_{i}=F(Z_{i})=F(\alpha +\beta X_{i}+\mu )=\frac{1}{1+e^{-Z_{i}}}=\frac{1}{1+e^{-(\alpha +\beta x_{i})}}$$ (1)

$$\log\frac{p_{i}}{1-p_{i}}=Z_{i}=\alpha +\beta x_{i}+\mu$$ (2)

#### 2.3.2 Conditional logit model

Conditional logit models are widely employed in location-selection problems, and they can be constructed drawing on two perspectives. First, a conditional logit model is used to construct a decision model for the older adults’ choice of the province. Assuming that older adults will consider a series of alternative provinces in their decision-making, the choice concerning which province to move to can be expressed as follows:

$$U_{ij}=F(Y_{ij}),\;(i=1,\cdots ,N;j=1,\cdots ,J)$$ (3)

where the individual utility is denoted by *U*_*ij*_, *F(Y*_*ij*_*); this* signifies the specific form of the utility function; *Y*_*ij*_ denotes the province characteristics; *i* signifies the number of older adults; and *j* denotes the number of provinces. When the older adults choose province *j* instead of province *k*, then *U*_*ij*_ > *U*_*ik*_, and *∀j* ≠ *k* stands true. At this point, the probability of choosing to move to province *j* can be expressed as follows:

$$P({\mathrm{chosen}}_{ij})=\frac{\exp[F(Y_{ij})]}{\sum_{j=1}^{J}exp[F(Y_{ij})]}$$ (4)

where *chosen*_*ij*_ is the variable of the province selected and is taken as 1 when selected and 0 when not.

Second, individual characteristics are included in the model to assess the older adults’ choice of provinces from the perspective of individual heterogeneity. In the conditional logit model, incorporating individual characteristics directly into the regression is not possible, as doing so would engender the problem of covariance. Therefore, the effect of individual characteristics is considered by establishing an interaction term with the province characteristics, and then the probability of province selection is as follows:

$$P({\mathrm{chosen}}_{ij}=1)=\frac{\exp[F(Y_{ij},\;W_{ij})]}{\sum_{j=1}^{J}\exp[F(Y_{ij},\;W_{ij})]}$$ (5)

where *W*_*i j*_ = *X*_*i*_×*Y*_*ij*_ denotes the interaction between individual features and province-related features.

### 2.4 Variables

Older adults’ selection process for a place of relocation can be broadly divided into two stages: the decision to relocate and the search for a home. In most cases, older adults tend to be less satisfied with their current residence because of environmental triggers, and after evaluating the feasibility of relocation, they choose to move if they feel that the benefits of relocation outweigh the costs and if they are convinced that relocation will bring greater satisfaction [29]. The sequence between the decision to move and the search for a home is not unique. Some older adults already obtain information from their family and friend networks about the environment they wish to relocate to and have examined the desired relocation destination in advance; hence, the search for a home occurs before the decision to move [30]. Regardless of the order of occurrence, this decision-making process is the result of a combination of factors that have been analysed in the study primarily at three levels: individual socioeconomic attributes, family ties, and regional characteristics (Table 1).

**Table 1:** Selection and source of independent variable indices.

Variable		Symbols	Description
Individual Level	Sex	GEN	Male = 1, Female = 0
	Age	AGE	> 75 years old = 1, ≤75 years of age = 0
	Level of education	EDU	Elementary school and below = 1, middle/high school = 2, college and above = 3
	Health Conditions	EHTH	Good physical health = 1, poor physical health = 0
	The main source of livelihood	MSOL	Support from other family members = 1, labor income/retirement pension = 2, other = 3
	Urban and rural classification	UARC	Current residence in urban area = 1, current residence in rural area = 0
Family level	Marital Status	MS	With spouse = 1, without spouse = 0
	Adult children	AC	Migrating into a household with adult children = 1, no adult children = 0
	Children ≤6 years of age	CH	Children aged 6 and under in the family migrating in = 1, no children aged 6 and under = 0
Regional Level	Annual temperature difference (°C)	TEMP	The temperature difference between January and July in the capital city of the province where the older adults are migrating to
	Annual sunshine hours (h)	ASH	Total sunshine hours from January to December in the capital cities of the provinces where older adults are migrating to
	Average annual precipitation (mm)	PRECIP	Average January–December precipitation in the capital cities of the province where the older adults are migrating to
	The average wage of employees (yuan)	MS	The average amount of money wages received by each employee of enterprises, institutions, and agencies in the province of current residence within a certain period
	Number of hospital beds (beds)	BED	Number of hospital beds per 10,000 population in the province of current residence
	Distance (h)	DIST	Minimum travel time between the current residence and the capital city of the province where the older adults are migrating to

Note: Because of the differences in question settings for the variables characterizing individual attributes in different years of the survey, corresponding processing is required to ensure data comparability. ①For the education level, the options of ‘not attended school’, ‘literacy class’ and ‘elementary school’ are combined into elementary school and below, ‘junior high school’, ‘high school’, ‘junior college’ and ‘general high school’, then the education level is considered to be junior high school/high school, and the options of ‘college specialist’, ‘college undergraduate’, ‘graduate’ and ‘graduate and above’ are college and above. ②In terms of health status, ‘healthy’ and ‘basically able to guarantee normal life and work’ are combined to form the term ‘good health status,’ whereas ‘cannot work normally or live on their own’, ‘cannot say’, ‘unhealthy but can live on their own’, and ‘cannot live on their own’ were combined to form the term ‘poor health’. The exploration for 1995–2000 affects the older adults’ migration choice variables when the health status data is lacking because the fifth census in 2000 excluded the health status survey. ③In the main source of living, ‘pension’, ‘labor income’, and ‘retirement and pension’ are coded as labor income/retirement pension, and ‘minimum subsistence allowance’, ‘living expenses for layoff’, ‘living expenses for internal retirement’, ‘minimum subsistence insurance’, ‘receiving basic living expenses’, ‘property income’, ‘insurance’, ‘unemployment insurance’, and ‘other’ are combined as other. ④Finally, for marital status, the options of ‘unmarried’, ‘divorced’ and ‘widowed’ are combined into the group of no spouse, while ‘spouse from first marriage’, ‘spouse from second marriage’ and ‘spouse’ are combined into the group of a spouse.

#### 2.4.1 Individual level

The investigation of specific factors was among the initial steps taken during this research, which also referred to the population migratory mobility theory. A sound state of physical and mental health is a crucial catalyst of migration in later life. This health-driven theory asserts that older adults with poor health are constrained by their environment, and they choose, often passively, to age in one place. Hence, they are less likely to move than their counterparts who have the advantage of good health [31]. Additionally, empirical research in some other countries has revealed that substantial differences exist among subgroups of older migrants, with women generally exhibiting a higher inclination to move and an increase in that propensity with age [32]. Further, older adults’ probability of migration increases with their education level [33]. Older adults with high and low incomes are more likely to migrate than those in the middle-income group. Older adults with higher incomes can choose more comfortable destinations, whereas older adults with low incomes are more likely to experience passive migration due to the increasing cost of living [29, 34]. Therefore, in this study, sex, age, level of education, health status, and primary source of livelihood were employed to measure the effects of personal attributes on the migration decisions of older adults.

#### 2.4.2 Family level

Interpersonal networks in Chinese society comprise close and distant relationships that are based on what is referred to as blood ties, and these relationships may change relative to time, space, and personal characteristics [19]. Moreover, in China, children are the primary providers of resources and support services for older adults, and the older adults’ social networks are predominantly family-linked, with the spouse and children being the foci. Therefore, family-related factors play a prominent part in influencing older adults’ migration in China [20, 35]. Additionally, the influence of family-related factors on the later-life migration of older adults is mainly driven by the latter’s search for intergenerational support. The movement of grandparents is influenced by the proximity of their grandchildren to their parents and whether there are newborns and young children who require care in their children’s families [36]. To assess how household-level determinants influence migration decisions of older adults, we took into account the presence or absence of adult children and preschool children aged ≤ 6 years in the household that would receive the migrants.

#### 2.4.3 Regional level

The geographical characteristics and appeal of the destination are critical factors that influence older adults’ migration-related choices. This encompasses both physical environmental variables, such as climate and ecology, and soft environmental factors, such as local finances, availability of public services at the local level, and opportunities for social interactions. Older migrants in developed regions/countries, such as Europe/the US, exhibit significant ‘heliotropism’. Retirees prefer to migrate over large distances to sunny, pleasant locations as their last destinations [12, 37]. Migration among older adults is also affected by the overall economic condition that obtains at a given time; in fact, this is a significant driver of migration. The consumption levels of locations and cost of living are the primary economic considerations of older adults. Since the cost of living is lower in areas with lower income levels, moving to lower-income areas turns out to be beneficial for older adults to reduce their cost of living [38]. Further, the demand for care grows as older adults’ health deteriorates, and the availability of medical services plays a key role in their choice to migrate [39]. Finally, the geographic distance factor can also be a key determinant, one way or the other, when older adults make choices concerning migrating to a place other than where they reside at present [40]. In the present study, the differences in environments between the migration destination and the current residence of older adults were compared in terms of the average employee wage, number of hospital beds, average annual precipitation, and annual temperature differences between the capital cities in the provinces where the potential for migration from other places is high.

The multicollinearity between variables in the binary logit model was evaluated, and it was found that the variance inflation factor values of the independent variables in each year were < 3, and no significant cointegration problem was observed.

## 3 Results

### 3.1 Basic Characteristics of the older migrants

#### 3.1.1 Chinese older migrants are mainly low-aged and married female

Overall, a study of the socioeconomic attributes of older migrants (Table 2) revealed that there was no significant change in the socioeconomic attributes of the older migrants between 1995 and 2015. First, the older migrants were predominantly female, except for the period between 2000 and 2005, when the older migrants were slightly more male than female. Second, the proportion of older migrants who were ≤75 years was significantly higher than that of those who were >75 years, with an annual difference of approximately 50 percentage points. Finally, in terms of marital status, there were many more older adults with spouses than older adults without spouses, with the proportion of older adults with spouses in the total population of migratory older adults in China approximately 70% across all years. These straightforward data comparisons indicate that the younger, female, spouse-bearing group dominates Chinese inter-provincial migrant older adults.

**Table 2:** Basic demographic characteristics of the older migrants.

Characteristics	Year	1995–2000	2000–2005	2005–2010	2010–2015
Sex (%)	Male	48.8	50.1	48.6	48.8
	Female	51.2	49.9	51.4	51.2
Age (%)	≤75 years old	78.7	76.4	74.8	76.8
	> 75 years old	21.3	23.6	25.2	23.2
Marital status (%)	With spouse	67.2	70.6	70.8	74.5
	Without spouse	32.8	29.4	29.2	25.5

#### 3.1.2 Social reasons: Primary drivers for China’s older migrants

**Fig 1:**
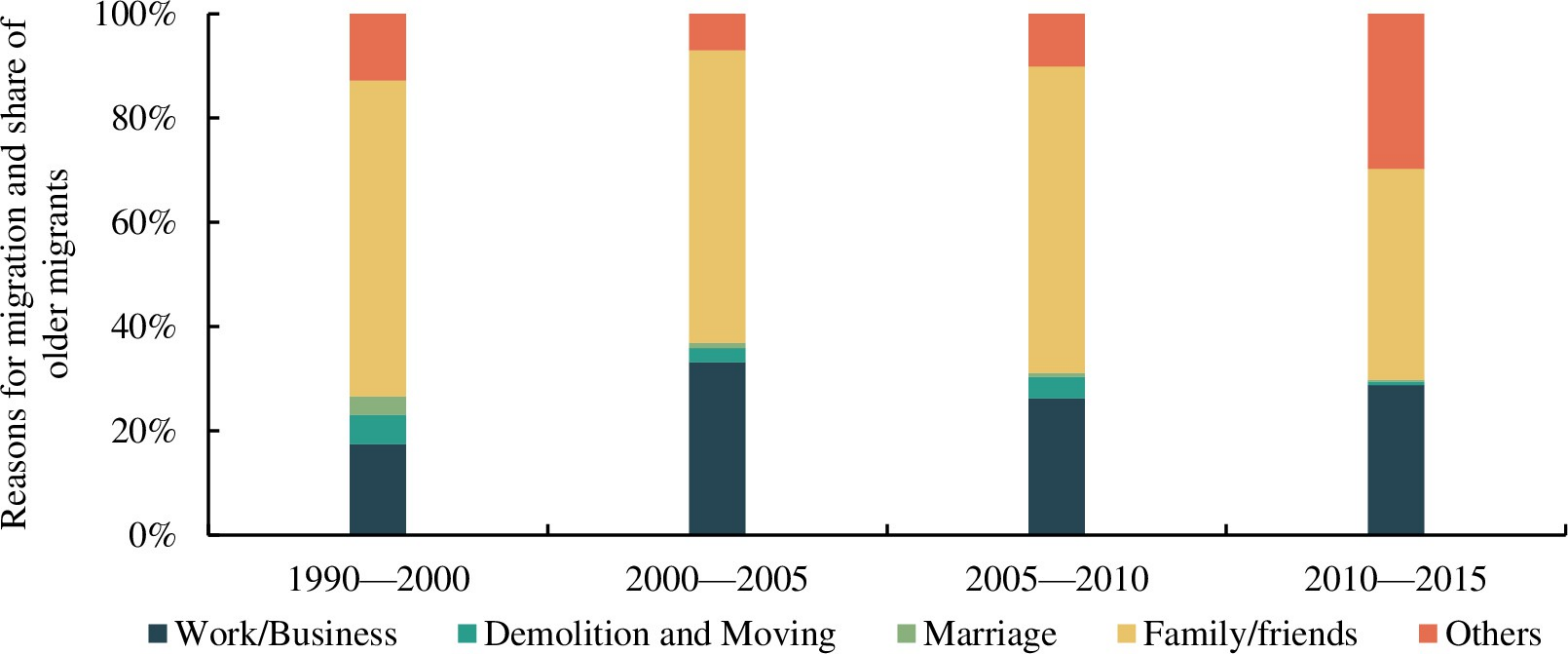
Reasons provided by older migrants for leaving their registered residences.

The survey data on the reasons for the migration of older adults in each period (Fig 1) revealed that ‘family/friends’ accounted for the highest proportion of the various self-reported reasons for inter-provincial migration among older migrants. The proportion of older adults who migrated because of family and friends was 60.558%, 56.084%, 58.836, and 40.547% of the total respondents in the four periods, respectively. This indicates that the migration behaviour of a majority of older adults is not driven by economic considerations and that the reasons for the migration of older adults and those of the young and middle-aged labour force differ significantly. Social reasons such as living close to family members, relatives, and friends constitute the primary motivation for China’s older migrants.

### 3.2 Migration Patterns of the older adults

#### 3.2.1 Older migrants primarily go to the eastern region and primarily leave the central region

Using the migration efficiency proposed by Bell et al. [41], we analyzed the differences in the migration of older adults between provinces in China (Table 3). It can be seen that from 1995 to 2015, the number of provinces with positive older migration efficiency was 13, 10, 14, and 10, respectively, all of which are slightly lower than those with negative older migration efficiency. The percentage of provinces with positive older migration efficiency located in the eastern region reached 69.23%, 70.00%, 64.29%, and 90.00% in the four periods, respectively. This indicates that the eastern region absorbs most of the older migrants in China and is the main destination for them. In contrast, the area in the center of the figure is significantly concentrated with provinces that have a negative older migration efficiency. All provinces in the central region had negative migration efficiency of the older adults during the period under study, except for Hebei Province which had a positive migration efficiency of the older adults between 1995 and 2000. The central region is the primary area from where older migrants leave the country, except for Yunnan, Ningxia, and Xinjiang, which also exhibited a negative older migration efficiency throughout the period under study.

**Table 3:** Changes in net migration of older adults in different provinces of China from 1995 to 2015.

Years	1995–2000	2000–2005	2005–2010	2010–2015
Eastern Region				
Beijing	65.96	69.40	70.91	58.36
Tianjin	5.88	95.60	62.79	39.68
Liaoning	26.09	11.83	3.53	-18.37
Shanghai	30.91	72.61	57.14	40.77
Jiangsu	31.51	-28.14	24.80	11.02
Zhejiang	3.70	6.31	45.79	32.11
Fujian	33.33	-11.11	34.48	27.62
Shandong	46.03	-49.80	-33.33	0.94
Guangdong	96.15	82.03	73.01	41.79
Hainan	-20.00	44.19	60.00	63.08
Central Region				
Hebei	20.69	-65.41	-16.88	-33.74
Shanxi	-33.33	-30.23	-24.32	-69.81
Jilin	-29.03	-40.00	-36.36	-36.84
Heilongjiang	-49.25	-82.04	-71.43	-74.84
Anhui	-55.56	-57.45	-59.49	-41.99
Jiangxi	-56.25	-74.65	-72.00	-47.67
Henan	-80.00	-82.48	-67.90	-38.04
Hubei	-14.29	-46.94	-38.89	-34.72
Hunan	-57.14	-72.09	-52.00	-19.69
Western Region				
Inner Mongolia	-15.00	-38.46	6.38	-2.78
Guangxi	-45.45	-34.43	28.00	-9.33
Chongqing	-23.08	-51.06	-15.00	-3.45
Sichuan	-43.33	-60.00	-36.14	-15.32
Guizhou	-16.67	-41.94	-33.33	-3.22
Yunnan	33.33	66.67	31.03	17.86
Shaanxi	-14.29	-29.03	-25.71	-22.22
Gansu	-58.33	-55.88	-56.25	-48.84
Qinghai	-80.00	-10.00	0.00	-100.00
Ningxia	33.33	50.00	12.50	-72.73
Xinjiang	20.83	30.43	34.88	8.91

#### 3.2.2 Increasing concentration of older adults migrating into urban areas by destination

**Fig 2:**
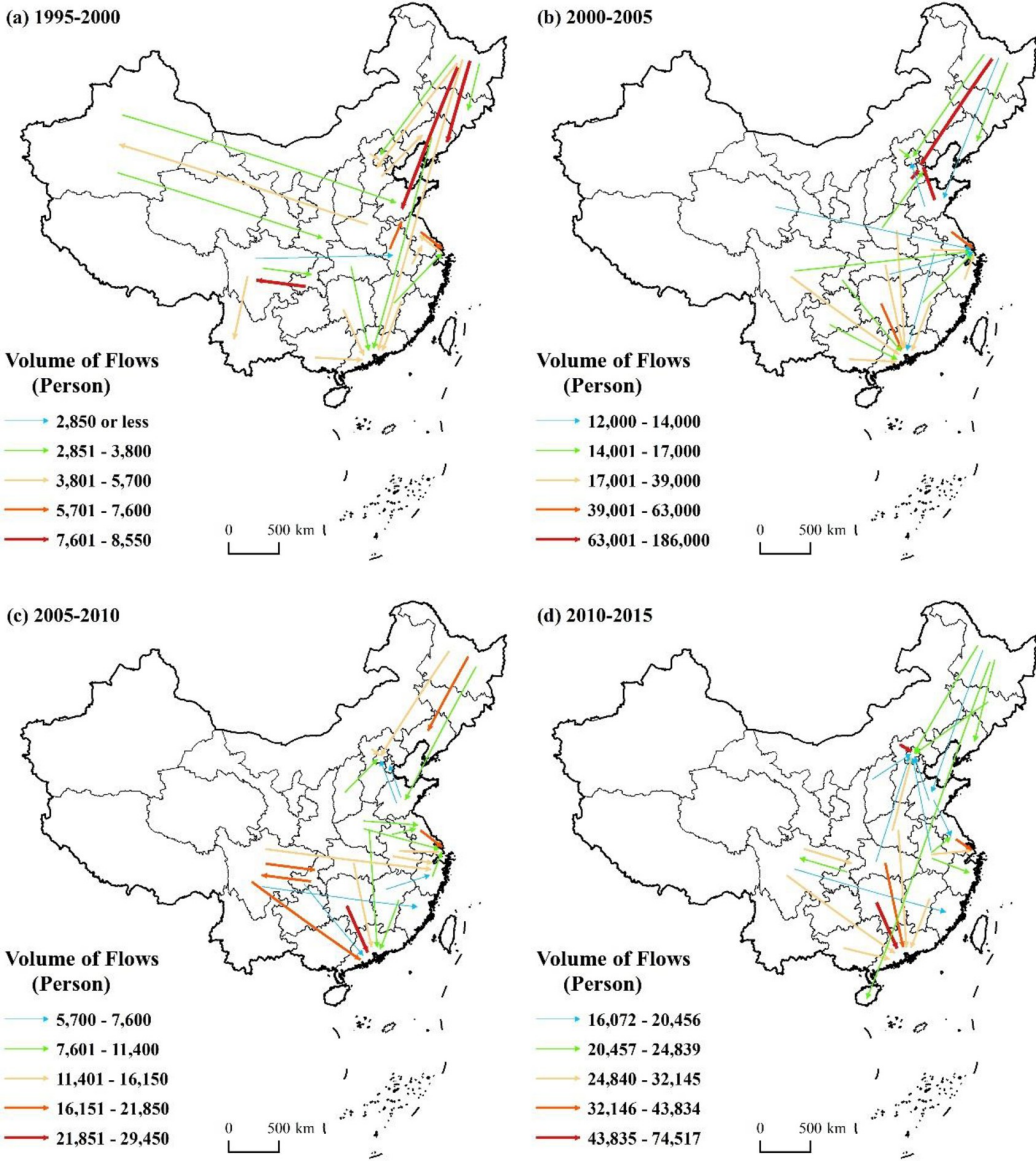
The 25 largest migratory flows of older adults to urban areas from 1995 to 2015. Figures are drawn by the authors according to the standard map of the National Surveying and Mapping Geographic Information Bureau (Approved drawing number: GS (2016)2893) (http://bzdt.ch.mnr.gov.cn/). All maps on this website are available for free download without copyright.

The top 25 older migratory flows to urban areas in China from 1995 to 2015 are shown in Fig 2, accounting for 38.98%, 52.69%, 34.94%, and 36.60% respectively of the total number of older adults migrating to urban areas. From 1995 to 2000, a total of 14 destination provinces were included in the top 25 older adult migratory flows. Among these, the largest migratory flows moved into the Guangdong province. Six migratory flows from different provinces moved to Guangdong; following closely were Shanghai and Beijing into which three and two flows moved from different provinces, respectively. Between 2010 and 2015, the number of destinations provinces in the top 25 older migratory flows had shrunk to 11, while the number of migratory flows to Beijing, Guangdong, and Shanghai had reached eight, six, and two, accordingly. Although Chinese older adults are not experiencing the same mass migration to the sunbelt as in Western countries, the migration pattern of older adults during 1995–2015 shows a clear latitudinal zonal pattern, i.e., the migration from the north (higher latitudes with colder climate) to south (lower latitudes with milder climate) is increasing, and the trend of concentration is becoming more and more significant. The urban areas in the Pearl River Delta, Yangtze River Delta, and Beijing-Tianjin-Hebei region, such as Beijing, Shanghai, and Guangdong, are becoming more attractive to older adults and are becoming their preferred migration destinations, and the trend of “stronger is stronger” is becoming more obvious. The one-child generation is now progressively entering the labor market in China (the rigid one-child policy was implemented in the late 1970s). They typically look for employment in major cities due to the fully developed labor market in metropolitan regions of economically developed provinces [21]. As traditional Chinese society is still based on the family as the basic organizational unit [20]. In the absence of a well-developed retirement system at the social level, migration to the cities where children live for daily care and to take care of grandchildren has become the norm, thus synchronizing the spatial patterns of migration of older adults with those of the labor force. Beijing, Shanghai, and Guangdong, which are the major provinces for young labor migration, are naturally the main destinations for older migrants [21]. It is worth noting that between 2010 and 2015, there was a long-distance migration from Heilongjiang to the urban areas of Hainan, which further indicates that older adults are gradually putting forward higher requirements for the climate and environment of the retirement destination, and the “migratory bird” retirement model of choosing more comfortable climate and environment destinations is gradually emerging [42].

#### 3.2.3 Older adults are less likely to want to move to rural areas, and their destinations are more dispersed

**Fig 3:**
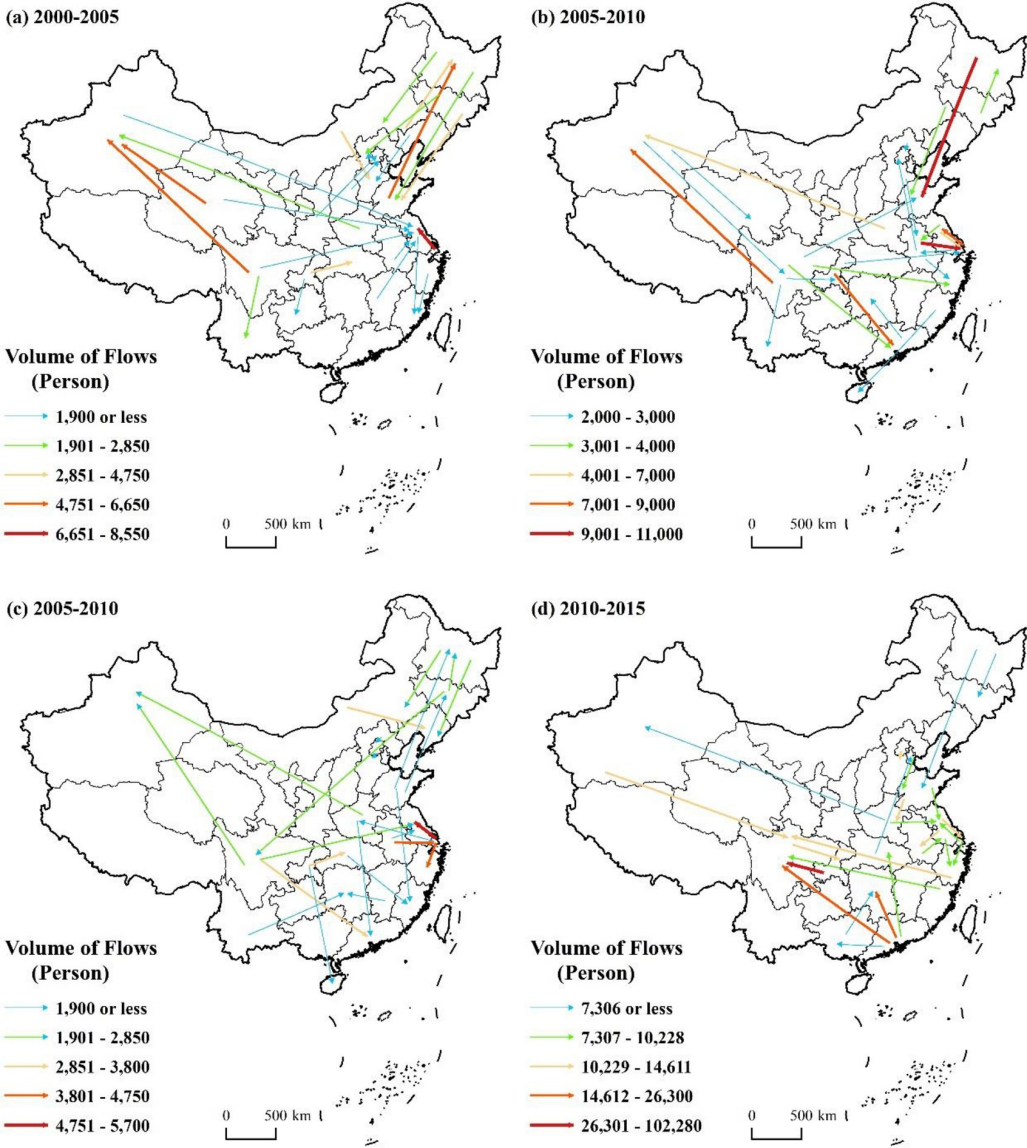
The 25 largest migratory flows of older adults to rural areas from 1995 to 2015. Figures are drawn by the authors according to the standard map of the National Surveying and Mapping Geographic Information Bureau (Approved drawing number: GS (2016)2893) (http://bzdt.ch.mnr.gov.cn/). All maps on this website are available for free download without copyright.

Fig 3 shows the 25 largest migratory flows of older adults to rural areas in China during the period between 1995 and 2015, accounting for 58.11%, 40.43%, 40.00%, and 32.10% of total migration to rural areas, respectively. First, there are significantly fewer older migrants migrating to rural regions than there are migrating to urban ones. The cumulative number of older migrants that moved to rural areas from 1995 to 2015 was 1,467,700, while the cumulative number of older migrants that moved to urban areas was 3.35 times higher (up to 4,913,900). The older adults of China migrate differently from that of industrialized nations like Europe and the United States, where older adults choose or are eager to relocate to more prosperous metropolitan regions rather than huge cities. In addition to wanting to spend more time with their children, the new generation of older folks is starting to prefer urban places and are becoming more accepting of the noisier metropolitan environment, particularly urban communities with good greenery, convenient outdoor exercise facilities, elevators, rich cultural and recreational activities and a good environment for neighborhood interaction. Easy accessibility to medical services is also an important reason for their pursuit of urban living [7–9]. Thus, the migration pattern of Chinese older adults is showing a gradual geospatial clustering in the urban areas of the Yangtze River Delta, Pearl River Delta, and Beijing-Tianjin-Hebei.

Second, the spatial features of the provinces where senior migrants are migrating to rural regions and that of those where older migrants are migrating to urban areas are very different. Compared with those who migrated to urban areas, those who migrated to rural areas were more dispersed and diversified in their choice of destination, with the number of destination provinces in the top 25 migration streams of older adults to rural areas in the four periods reaching 11, 16, 18 and 15, respectively. Among the top 25 older migratory flows to rural areas between 1995 and 2015, those with larger older migrants were mainly from more developed provinces to provinces with lower levels of economic development. The more typical ones were migration from Shanghai to Jiangsu, Guangdong to Sichuan, and Sichuan to Xinjiang. Provinces with a strong economy also typically have superior natural features, more employment opportunities, and higher income levels. While preventing their own people from leaving, these provinces also draw a lot of employees from other provinces, and older adults frequently move to such locations when they are young and looking for job prospects. As workers age, their ties to their birthplace significantly impact their migration decisions. After leaving their hometowns for work at a young age, many people choose to return to their hometowns to live and retire; this has become a common migration pattern known as “return migration”. A small number of economically developed provinces retain most of the migrating population, while the outflow provinces are widely distributed; this is why the destination choice of the older adults migrating to rural areas is more dispersed than that of the older adults migrating to urban areas.

### 3.3 Decision-making mechanisms underlying migration of older adults

#### 3.3.1 Results of the binary logit model

In this study, whether migration occurred after the age of 60 years was the dependent variable (migrated = 1, not migrated = 0), and individual, household, and regional characteristics were the independent variables for the binary logit regression model employed to explore what kind of older adults are more likely to migrate. The results of this analysis are presented in Table 4.

**Table 4:** Results of the binary logistic model.

Model I				
Years	1995–2000	2000–2005	2005–2010	2010–2015
Personal factors				
GED (Female as the reference group)	-0.020	-0.097**	0.152**	0.157***
AGE (≤75 years old as the reference group)	-0.415***	-0.218***	-0.475***	-0.853***
EDU (Primary school and below as the reference group)				
Middle School/High School	0.980***	0.450***	0.487***	0.318***
College and above	1.276***	0.912***	0.879***	0.524***
EHTH (with poor physical health as the reference group)	—	0.420***	0.569***	0.708***
MSOL (Other members of the family provide as the reference group)				
Labor income/retirement benefits	-0.235**	-0.203***	-0.299***	-0.317***
Others	-0.225	-0.438***	-0.678***	-0.551***
Family Factors				
MS (without spouse as the reference group)	-0.064	-0.032	-0.040	-0.067
AC (without adult children as the reference group)	-0.161*	0.728***	-0.114*	-0.474***
CH (without children under 6 years old as the reference group)	0.252**	0.117**	0.590***	0.808***
Regional Factors				
TEMP	1.130***	0.206*	-0.761***	-0.846***
ASH	-0.479***	-0.363***	-0.292***	0.066
PRECIP	-0.391	0.441***	-0.244*	0.139
MS	2.569***	2.874***	2.276***	2.836***
BED	0.352**	-0.015	0.876***	0.506**
*Number of obs*	127118	312014	175687	288937
*Pseudo R* ^*2*^	0.0524	0.0826	0.0788	0.0749

Note: *** indicates significant at the 1% level, ** indicates significant at the 5% level, and * indicates significant at the 10% level; non-standardized regression coefficients are reported in the table.

(1) Individual level

During the period 1995–2000, the migration decisions of older adults were not determined by the sex they belonged to. Between 2000 and 2005, older Females were more likely to migrate than older Males; however, this changed in the next two periods, with men increasingly becoming more likely to migrate than women. Regarding age, the probability of migration was consistently weaker for the population aged > 75 years than for the population aged ≤ 75 years. Considering the rising need for healthcare as individuals age and that social security and health insurance coverage are still primarily based on administrative borders in China, people choose to stay in the same place, which reduces their likelihood of migration [35]. When compared with the group of older adults with an education level of elementary school and below for whom the possibility of migration was the least, those with an education level of college and above were more likely to migrate, followed by those with an education level of junior high school. The general education level of older adults is on the rise as society changes. Additionally, they have richer travelling experience and a better understanding of modern and sophisticated transportation and communication systems. The increasing benefit of retirement security also equips them with the means to relocate to faraway places and seek out more liveable and alluring retirement locations; all this inevitably results in more long-distance migration [43, 44]. Regarding health status, older adults in good health have a higher probability of migration than those in poor health. Between 1995 and 2015, the regression coefficient increased from 0.420 to 0.569 and then to 0.708, indicating that the positive influence of physical health status on the migration decisions of older adults remains; rather, its importance as an influencing factor has increased. For older adults, physical health guarantees quality of life, and poor health frequently restrict their range of activities and motion. Moreover, migration may be very inconvenient for older adults who are ill and in need of medical attention. Therefore, older adults in excellent health are more inclined to move, whereas those in bad health often opt to age where they are already based [31]. Finally, the probability of older adults’ migration is high when their primary financial resource constitutes the incomes of other members of the household, as against their income or retirement pension. Although we did not directly ask the older adults the specific amount they earned, we found out which income source was the most critical and reflected their ability to financially support themselves. When other family members are the primary source of financial support for older adults, it indicates that they are more or less dependent on their family members and are more likely to choose to move to live with these family members.

(2) Family level

Among the family-level factors, the presence or absence of a spouse did not influence the decision to move in old age in any of the four periods. The presence or absence of adult children in the older migrant’s destination family was positively significant between 2000 and 2005, that is, the presence of adult children in the destination household promoted older migration. However, this factor was negatively significant during the other periods. This indicates that older adults are more reluctant to move when adult children are present in the household they are moving into. Compared with the presence of adult children, the presence of children aged < 6 years in the destination family had a more stable effect on the migration decisions of the older adults, consistently and positively contributing to their migration. Owing to the growth of the market economy in China, two-income families now struggle to find time to care for their kids. The Chinese, given their strong family orientation, are more at ease leaving their children in the care of their parents than in the hands of nannies (when childcare facilities are inadequate). Therefore, young couples frequently remain with their parents until the third generation of children reach adulthood or enrol in school, and the old population’s movement is strongly correlated with the necessity of the families to have the grandkids duly taken care of [21, 35, 36].

(3) Regional level

Regarding region-level attributes, only the average wage of workers in the potential migration location consistently influenced the migration decisions of older adults, and the four periods of them were positively significant. That is, the higher the wage level of employees in the potential migration location, the more willing older adults were to relocate there. The average wage of employees can, to some extent, reflect the level of socioeconomic development of a province, and in this case, the cost of living is a critical indicator of the financial ability of adult children and the quality of regional-level healthcare services for older adults [45, 46]. In China, older adults tend to move from smaller cities, towns, and rural areas where the cost of living is low to countries or regions where the cost of living is high. Possibly, this is so because most of them want to move closer to their children to take care of their grandchildren and receive care from their adult children. The temperature difference where they are and the potential migration region also exerted a positive and significant impact in the first decade but became negative and significant in the second decade. That is, between 1995 and 2005, older adults were more willing to migrate when the temperature difference between January and July in the potential migration region was higher; however, between 2005 and 2015, the probability of migration reduced if the temperature difference was too high. Further, average precipitation significantly and negatively affected older adults’ migration decisions between 1995 and 2010. For this period, it was found that where the average precipitation in the potential migration site was high, the probability of older adult migration to that place was low. The number of hospital beds also positively influenced older adults’ migration decisions during all three periods, and in the first and last five- and ten-year periods, when the number of hospital beds in the potential migration location was higher, migration became appealing to older adults. Finally, the effect of annual sunshine count on the migration decisions of older adults was the most variable, exerting different effects on the migration decisions of older adults at different times.

#### 3.3.2 Results of the conditional logit model

Table 5 presents the results of the conditional logit model with the addition of interaction terms between area attributes and rural-urban classification of destination. Furthermore, Model 2 examines the differences in the choice of residence between older migrants to urban areas and those migrating to rural areas. The results highlight the presence of differences in location-related decision-making between the older adults migrating to urban areas and those relocating to rural areas. First, from 1995 to 2000, compared with older migrants who moved to rural areas, those who chose to move to urban areas preferred destinations with more hospital beds, lower average annual precipitation, and fewer hours of sunshine per year, in addition to those at shorter distances from their current residence. Second, a slight change was observed between 2000 and 2005 in the residence preferences of older migrants who moved to urban and rural areas. Older migrants who chose to move to urban areas continued to prefer destinations with lower average annual precipitation and those at shorter distances from their current residence. However, higher average wages discouraged this group from migrating to rural areas, that is, the older adults who chose to move to rural areas chose to live in areas with lower average wages. From 2005 to 2010, a period in which only the average employee wage coefficient was significant, older migrants who chose to move to urban areas were positively influenced by the average employee wage in the area they moved to, with a higher average regional employee wage increasing the likelihood of their migration. Finally, from 2010 to 2015, the older adults who chose to move to urban areas were more likely to move to provinces with higher annual sunshine hours and average employee wages, while those who chose rural areas were more likely to move to provinces with higher average annual precipitation and more hospital beds and those at longer distances from their current residence. The possible reason for the insignificant influences in the model could be that these factors are equally critical in determining the choice of location, both for the older adults who chose to move to urban areas as well as for those who chose to move to rural areas.

**Table 5:** Results of the conditional logit model.

Model II								
Years	1995–2000		2000–2005		2005–2010		2010–2015	
TEMP	0.338	0.617	0.966***	0.634**	0.362**	0.022	-0.078	0.017
ASH	0.225**	0.909***	0.616***	0.950***	0.384***	0.442***	0.360	0.680***
PRECIP	1.134***	2.007***	1.796***	1.301***	1.330***	0.941**	1.059	0.669***
MS	-0.063	-0.401	-0.594***	0.326	0.870***	0.108	0.478	-0.061
BED	-0.235*	-0.678**	0.477***	0.291	-0.806***	-0.400	0.103	1.473***
DIST	-0.138***	0.033	-0.478***	-0.171**	-0.250***	-0.154*	-0.289	-0.158***
Interaction items:								
* Migration to a town								
TEMP*		-0.390		0.462		0.439		-0.166
ASH*		-0.975***		-0.409**		-0.067		-0.442***
PRECIP*		-1.167*		0.516		0.486		0.583**
MS*		0.541		-1.058***		0.936*		0.783***
BED*		0.588*		0.179		-0.520		-1.962***
DIST*		-0.241**		-0.342***		-0.118		-0.195***
*Number of obs*	503×30		2068×30		1035×30		1953×30	
*Pseudo R* ^*2*^	0.0137	0.0204	0.0801	0.0869	0.0235	0.0252	0.0206	0.0247

Note: *** indicates significant at the 1% level, ** indicates significant at the 5% level, and * indicates significant at the 10% level; non-standardized regression coefficients are reported in the table.

## 4 Discussion and conclusion

Concerning migration among the Chinese population, academic attention has tended to primarily focus on the migration of the labour population, relatively neglecting older migrants. In recent years, in tandem with population ageing and increasing migration, the number of older migrants in China has gradually increased. To address the demand for practice, academics have gradually begun to study migration among older adults; however, most studies concerning them have worked with single-period data or only studied the migrants of a specific region. It is not possible to compare the basic characteristics of older migrants, including spatial patterns and selective mechanisms across time, and understanding the situation of this group at the national level is difficult. Further, considering older migrants as a whole enables the description of the broad patterns of older adults’ migration but masks significant differences within subgroups. Therefore, in this study, we utilised microdata pertaining to four periods from the 2000 and 2010 population censuses and the 2005 and 2015 national 1% population sample surveys to classify older migrants into two categories at the national level—migrants to urban areas and migrants to rural areas.—Further, we examined their cross-provincial migration patterns and what explains their choices.

Previous research has demonstrated that population ageing is usually accompanied by the onset of chronic diseases. When health conditions are poor, ageing in the place where one is already based is the best option, and the likelihood of relocation is greatly reduced [22, 31]. From a sex-based perspective, older Chinese men prefer to be more independent, while their female counterparts prefer to have something to fall back on, say, their spouse and/or their children [47]. The findings of the present study indicate that China’s present-day older migrants are predominantly young, female, and married. Their self-reported reasons for migration revealed that a majority of the older migrants relocated for reasons related to family members, relatives, and friends.

Regarding migration efficiency, the present study found that between 1995 and 2015, the number of provinces with positive migration efficiency for older adults was less than that of provinces with negative migration efficiency. The eastern provinces have attracted many older migrants, while the central region has seen a large outflow of older migrants. Further, by dividing older migrants into those migrating to urban areas and those to rural areas, it was observed that between 1995 and 2015, the number of older adults migrating to urban areas was much higher than that of those migrating to rural areas. For older migrants who chose to move to urban areas, economically developed provinces such as the Pearl River Delta, Yangtze River Delta, and Beijing-Tianjin-Hebei region (which includes Beijing, Tianjin, and Shanghai) were the most attractive. Simultaneously, a growing trend was observed among older migrants of migration from colder regions at high latitudes to low-latitude regions with milder weather, even as older adults’ choices concerning retirement destinations became more diversified and responsive to their own needs. By contrast, those older migrants who chose to move to rural areas were more dispersed in their choice of destination in terms of spatial distribution, and they primarily moved from more developed provinces to relatively less developed ones.

Thus, China’s older adults have a migratory pattern that is very different from that of older adults in Western industrialised nations who are quite eager to move from urban to rural regions [4, 6]. A majority of older migrants in China are ‘centripetal migrants’, that is, they move from rural to urban areas. Most Western nations have developed insurance and pension systems, with social pensions serving as the primary source of income post retirement In the Western cultural context, older adults have relatively lower expectations from and dependence on their children while they are ageing. Older adults have more free time after retirement, and to make the most of their later years, a great majority of them opt to retire to rural locations having beautiful natural settings and clean air [12, 48]. The relatively harsh Chinese one-child policy, which was implemented in the late 1970s, required households to have just one child. This one-child generation, which is now progressively entering the workforce and is often well-educated and has a broader outlook, frequently seeks work in urban areas where the labour market is fully developed [21]. It is very common for retired parents to move to places where their children have found employment so that the former can take care of their grandchildren.

Among all of the factors that influence the migration decisions of older adults, the present study found that, at an individual level, older adults aged ≤ 75 years consistently had a higher probability of migration than those aged > 75 years. The higher their education level, the more the likelihood that they would migrate: older adults with an education level of primary school and below being the reference group in this case. Older adults who went to college and beyond had the highest probability of migration, followed by those who studied till junior high school and high school. Those with an education level of elementary school and below lagged on this front. Older adults in poor health were less likely to migrate than those in good health. Finally, the likelihood of migration was also higher when the main financial resource for older adults was the income(s) of other members of the household than when it was their income or a severance pension. At the household level, the older adults’ decision to migrate was positively and significantly influenced by the presence of children aged ≤ 6 years in the destination household. In other words, the older adults were more inclined to migrate if there were children aged ≤ 6 years in the destination household. Finally, at the regional level, older adults were more likely to migrate when the potential migration destination was a metropolitan region where employees’ wages were on the higher side. We also noted the differences in locational preference between older adults who chose to move to urban centres and those who chose to move to rural areas, with the same influencing factors exerting different effects in different years when the interaction terms of regional attributes and the type of urban and rural destination were included.

Numerous studies have confirmed that women exhibit a higher propensity to migrate and that this propensity grows stronger with age [32]. The present study highlighted a very different aspect: Older adults in China had a lower propensity to migrate, and a person’s sex did not affect their migration decisions. Most current Chinese social security programs are still based on administrative boundaries, which may explain this finding. People tend to stay in areas where they can access medical care and social insurance, for migration would be very inconvenient given the increased demand for medical care as a person ages [35]. At the family level, it was found that spouses tended to influence older adults’ migration decisions; for example, older adults in the US are about twice as likely to relocate after being widowed than in the years before that [49]. However, for Chinese older adults, the presence or absence of a spouse did not exert any significant impact on their migration decisions. Notably, the probability of older adults’ migration was affected more favourably by the presence of grandkids than by the presence of adult children, and the probability of older adults’ migration decreased by the presence of adult children. This indicates that older adults migrate more frequently when intergenerational caring is a requirement and that the presence of grandchildren is a ‘stimulant’ for this movement. The phenomenon of intergenerational family transfer is a common one in most countries. In China, which is in the midst of transformation and change, the government’s pension system is not yet perfect. This coupled with the traditional culture of Confucian filial piety, children constitute a crucial safeguard for parents in their old age. However, adult children are more likely to provide financial help for their parents’ care than bring them to their homes for personal care [50]. That said, under the influence of the traditional family ethics of Chinese society, everyone in a traditional family adopts the logic of the ‘survival and reproduction principle’ from birth, and the Chinese are concerned with having more children and being more blessed as such. China’s long-standing patriarchal society has shaped people’s mindset of ‘having grandchildren’, and the modern family, which is born out of the traditional family, cannot help but uphold it. When fathers ask grandparents to take care of their grandchildren, the grandparents, who are influenced by deep-rooted family ethics, are conscious of their responsibility to nurture the grandchildren [51]. Thus, the presence of minors in the household is a greater incentive for older adults to migrate than the presence of adult children.

This study has some limitations. First, is the absence of an indicator of household registration nature owing to the abolition of the distinction between agricultural and non-agricultural households in China in 2015 and the unified registration of all properties as resident households. In China, the urban–rural dichotomy of ageing is conspicuous, and a majority of the older adults with rural household registration are located in rural areas and engaged in agriculture as their lifelong occupation. Hence, they have meagre savings and do not enjoy the retirement system. By contrast, most of the older adults in urban areas are not employed in the agricultural sector, have held jobs, and, therefore, enjoy retirement benefits. The differences in the nature of the study subjects affect their migration behaviour differently, and our failure to distinguish them in this study resulted in some bias in the findings. Second, due to the unavailability of certain data, some indicators that may influence the migration decisions of older adults, such as the provinces where the older adults are born and their regional economic attributes, were not duly considered as drivers of migration as far as the present study was concerned. This prevented a more detailed exploration of the impact of early life experiences on the migration decisions of older adults. Finally, migration was defined based on whether the same province was the usual and current residence five years ago, and the older adults may have moved to different destinations several times during this five-year period (which could not be taken into account). We recommend that future research should divide the category of older adults into more precise sub-categories, and the selection of variables should be so that more accurate and insightful results can be obtained.

## Supporting information

S1 Data (XLSX)
